# Retroperitoneal Abscess Due to Klebsiella pneumoniae Treated With Multiple Percutaneous Catheter Drainages in a Patient With Diabetes: A Case Report

**DOI:** 10.7759/cureus.93951

**Published:** 2025-10-06

**Authors:** Sinem Ülke, Sevil Uygun İlikhan, Canan Güneş, Pelin Yumuşak, Selma Karaahmetoğlu

**Affiliations:** 1 Department of Internal Medicine, Ankara Bilkent City Hospital, Ankara, TUR

**Keywords:** case report, diabetes mellitus, klebsiella pneumoniae, percutaneous drainage, retroperitoneal abscess

## Abstract

Retroperitoneal abscesses are rare but serious clinical conditions characterized by delayed diagnosis, insufficient drainage, and high morbidity. They are more common in immunocompromised individuals, such as those with diabetes mellitus.

A 60-year-old female with a history of type 2 diabetes mellitus presented with complaints of right flank pain, nausea, and vomiting. Laboratory tests revealed leukocytosis, and abdominal imaging identified a 10 cm abscess in the right retroperitoneal space. Percutaneous drainage was performed with a pigtail catheter under ultrasound guidance. *Klebsiella pneumoniae* was isolated from the abscess culture. Clinical improvement was observed during follow-up. The patient received meropenem and cefazolin for a total duration of four weeks. Upon clinical improvement, she was discharged.

Retroperitoneal abscesses often present with nonspecific symptoms, leading to delays in diagnosis. In patients with diabetes, the risk of severe infections with Gram-negative pathogens like *Klebsiella pneumoniae *is elevated due to impaired immune response. Early imaging, timely percutaneous drainage, and appropriate antibiotics are crucial for effective management.

Retroperitoneal abscesses should be considered in patients with diabetes presenting with abdominal pain and systemic symptoms. Prompt diagnosis and early intervention significantly reduce morbidity and improve outcomes.

## Introduction

Retroperitoneal abscesses are rare but potentially serious infections that are often difficult to diagnose and can lead to severe complications. Delays in diagnosis and inadequate drainage significantly increase morbidity and mortality. Due to the complex anatomy of the retroperitoneal space, infections in this region are typically recognized late. Anatomically, the retroperitoneum is divided into three compartments: the anterior pararenal space, the perirenal space, and the posterior pararenal space [1].

Imaging techniques such as computed tomography (CT), magnetic resonance imaging (MRI), and ultrasonography are widely used in the diagnostic process. These abscesses are most commonly associated with infections originating from the urinary or gastrointestinal systems. Bacterial spread from the urinary tract is the most frequent cause, while gastrointestinal sources, such as perforated appendicitis, colon cancer, diverticular disease, and Crohn’s disease, represent the second most common origin. Infections of bony structures, such as Pott’s disease and osteomyelitis, as well as hematogenous dissemination from distant septic foci, may also contribute to retroperitoneal abscess formation. Iatrogenic infections following abdominal or pelvic surgeries are additional, albeit less common, etiologies.

The majority of reported retroperitoneal abscess cases occur in immunocompromised individuals, including patients with diabetes, cirrhosis, malignancies, remote infections, chronic steroid use, and chronic kidney disease. In particular, recurrent urinary tract infections associated with diabetes predispose patients to infections that may extend into the retroperitoneal space. Early diagnosis and effective drainage are essential for successful management. The current case report presents the development and treatment of a retroperitoneal abscess in a patient with diabetes, emphasizing the importance of prompt intervention [2,3]. This case is reported due to its rarity and to highlight the clinical significance of *Klebsiella pneumoniae* as an uncommon causative pathogen of retroperitoneal abscesses in patients with poorly controlled diabetes mellitus.

## Case presentation

A 60-year-old female presented to the emergency department with complaints of fever, chills, nausea, vomiting, and right flank pain. Prior to admission, she experienced a syncopal episode following dizziness. Her medical history revealed intermittent dysuria for which she had been prescribed antibiotics by her primary care physician; however, the patient did not recall the name of the antibiotic and reported irregular adherence to the medication. She denied symptoms such as cough, recent weight loss, altered bowel habits, hematuria, tea-colored urine, steatorrhea, or jaundice. There was no history of trauma or abdominal surgery. The patient lived alone and had a known diagnosis of type 2 diabetes mellitus for the past 10 years.

On physical examination, her body temperature was 38.2°C. Notable right costovertebral angle tenderness was observed. Her body mass index (BMI) was 30 kg/m². Abdominal examination did not reveal tenderness in the right upper quadrant or a positive Murphy’s sign to suggest cholecystitis; there was no right lower quadrant tenderness, rebound tenderness, or guarding to suggest appendicitis. Laboratory findings are summarized in Table 1.

**Table 1:** Baseline and post-treatment laboratory results ↑ indicates values above the reference range; HPF: high-power field; CRP: C-reactive protein; ALP: alkaline phosphatase; AST: aspartate aminotransferase; ALT: alanine aminotransferase; GGT: gamma-glutamyl transferase

Parameter	Baseline Results	Post-treatment Results	Reference Range
Leukocyte	15.22 ×10⁹/L ↑	8 ×10⁹/L	3.9–10.2 ×10⁹/L
Hemoglobin	11.6 g/dL	11.1 g/dL	12–15.6 g/dL
Platelet	126 ×10⁹/L	329 ×10⁹/L	150–400 ×10⁹/L
CRP	96 mg/L ↑	3 mg/L ↑	0–5 mg/L
Procalcitonin	55 µg/L	0.06 µg/L	<0.16 µg/L
Creatinine	1.71 mg/dL ↑	1.01 mg/dL ↑	0.5–1.1 mg/dL
Urea	114 mg/dL ↑	72 mg/dL ↑	19–49 mg/dL
Sodium	133 mEq/L	135 mEq/L	132–146 mEq/L
Potassium	4.1 mEq/L	4.3 mEq/L	3.5–5.5 mEq/L
ALT	8 U/L	10 U/L	<35 U/L
AST	23 U/L	7 U/L	<50 U/L
ALP	240 U/L	108 U/L	53–141 U/L
GGT	110 U/L	47 U/L ↑	<38 U/L
Blood glucose	286 mg/dL ↑	130 mg/dL ↑	70–99 mg/dL
HbA1c	16 %	—	—
Ferritin	176 µg/L	—	10–291 µg/L
Leukocyte (urine)	162 /HPF	5 /HPF	—
Erythrocyte (urine)	1 /HPF	12 /HPF	—
Nitrite / Esterase	Positive / +3	Negative / trace	Negative
Blood culture	Negative	—	—
Urine culture	Negative	—	—
Abscess culture	*Klebsiella pneumoniae *(+)	—	—

**Figure 1:**
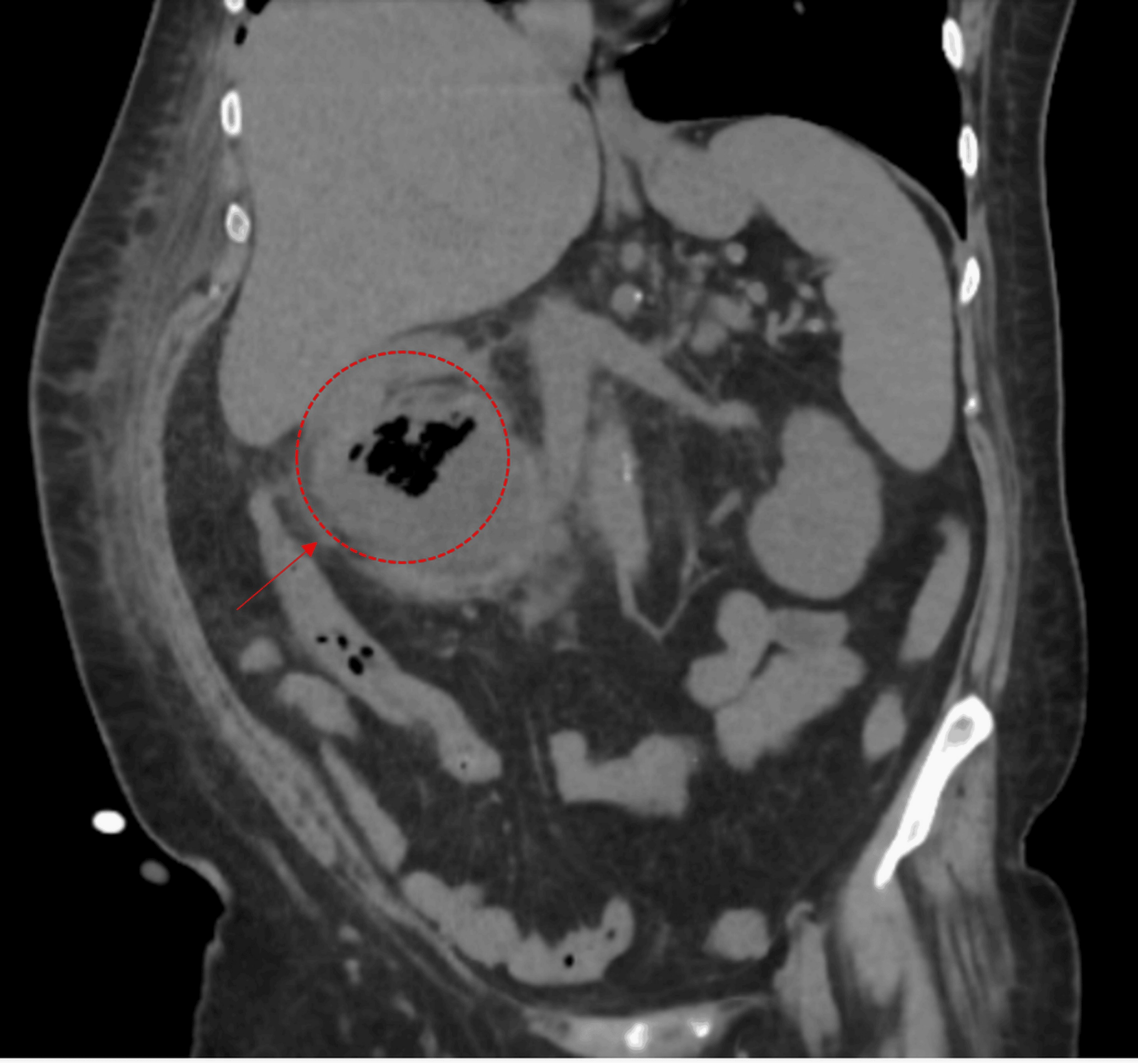
Contrast-enhanced CT image showing a large retroperitoneal abscess in the right renal fossa, extending into the psoas muscle, marked with a red arrow

**Figure 2:**
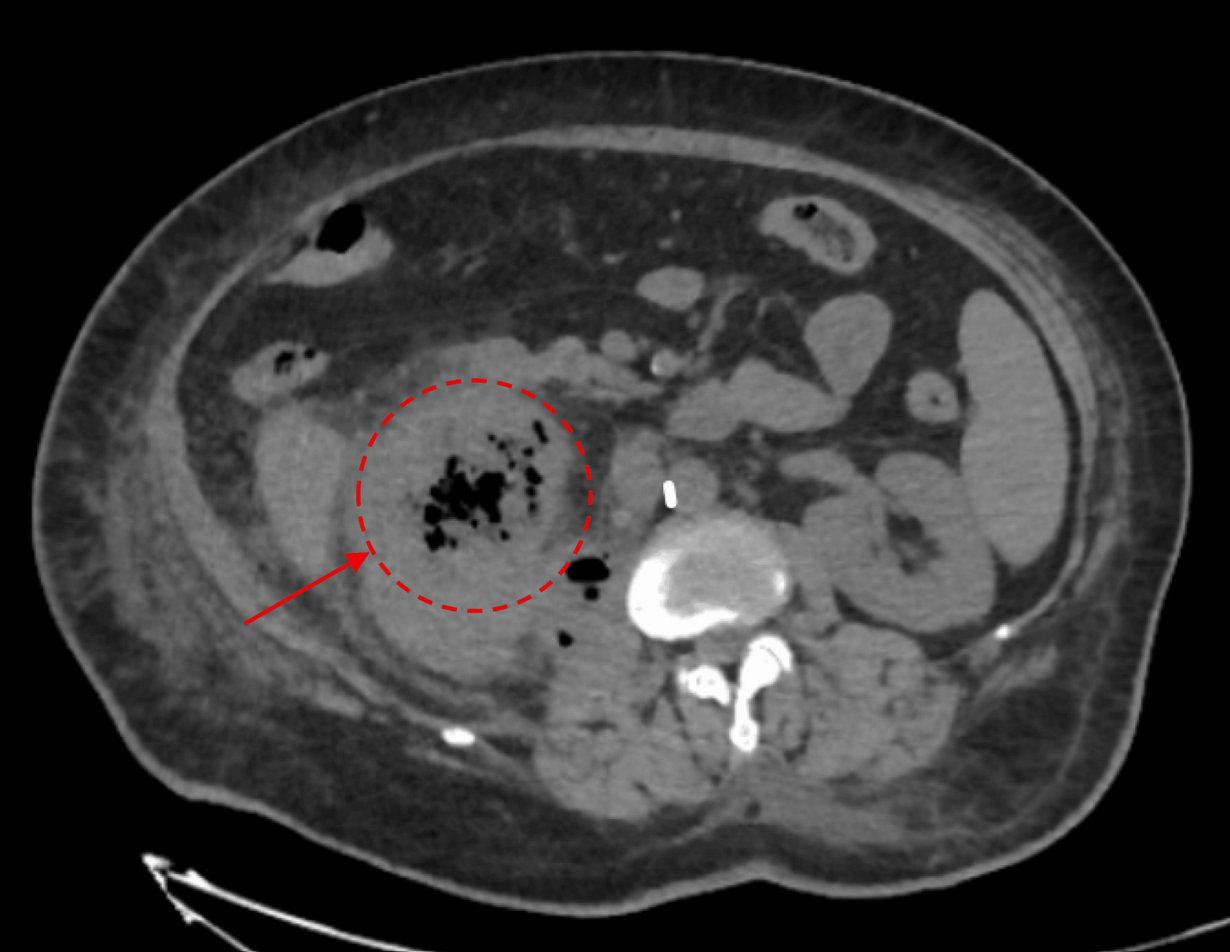
CT scan demonstrating the multiloculated nature of the retroperitoneal abscess (marked with a red arrow), consistent with the need for percutaneous drainage

On the fifth day of hospitalization, an abdominal CT scan revealed a 10 cm multiloculated abscess containing air in the right renal fossa, extending into the psoas muscle and renal parenchyma (Figures 1, 2). Both the interventional radiology and urology teams were consulted at this stage; the urology team did not recommend surgical intervention. On day eight, follow-up ultrasonography confirmed a persistent loculated abscess, and interventional radiology subsequently performed percutaneous drainage with placement of a pigtail catheter. At that time, the patient was receiving intravenous ceftriaxone; later, due to clinical status, the antibiotic regimen was escalated to meropenem and cefazolin, and drainage was maintained via the catheter. Because of persistent symptoms, a second drainage procedure was performed on day 14, resulting in both clinical and biochemical improvement. Cultures from two separate abscess drainage samples grew *Klebsiella pneumoniae. *The antibiotic susceptibility profile (antibiogram) of the *Klebsiella pneumoniae *isolate is demonstrated in Table 2.

**Table 2:** Antibiotic susceptibility profile of Klebsiella pneumoniae isolate

Antibiotic	Susceptibility	MIC (mg/L) / Zone Diameter (mm)
Ceftazidime	Resistant	≥32.0
Ciprofloxacin	Susceptible	≤0.06
Piperacillin/Tazobactam	Resistant	—
Ampicillin	Resistant	≥32.0
Cefuroxime	Resistant	≥64.0
Cefuroxime Axetil	Resistant	≥64.0
Ceftriaxone	Resistant	≥64.0
Amikacin	Susceptible	2.0
Trimethoprim/Sulfamethoxazole	Susceptible	≤20.0
Amoxicillin/Clavulanic Acid	Susceptible	≤4.0
Ertapenem	Susceptible	≤0.12
Ampicillin (oral)	Resistant	≥32.0

During follow-up, C-reactive protein (CRP) levels decreased from 270 mg/L to 3 mg/L, procalcitonin from 55 µg/L to 0.06 µg/L, and leukocyte count from 15.2 × 10⁹/L to 8.0 × 10⁹/L. Post-treatment laboratory results are presented in Table 1. The patient received meropenem and cefazolin for a total duration of four weeks. The rationale for including cefazolin in the treatment regimen was to optimize therapy according to the *Klebsiella pneumoniae* isolate’s susceptibility profile, which demonstrated sensitivity to first-generation cephalosporins; thus, once culture and sensitivity results were available, therapy was narrowed to avoid unnecessary broad-spectrum coverage. Upon clinical improvement, she was discharged. A follow-up ultrasound performed two weeks after discharge revealed no residual abscess. CRP was below 4 mg/L, and fasting blood glucose was 116 mg/dL, indicating good glycemic control. Regarding glycemic management, the patient had been on insulin therapy prior to hospitalization but reported irregular use and occasionally missed doses, leading to suboptimal control. During hospitalization, a structured basal-bolus insulin regimen was implemented, and at discharge, the patient was advised to continue insulin therapy under closer monitoring with reinforced education on adherence.

## Discussion

The diagnosis of retroperitoneal abscesses is often delayed due to their nonspecific initial symptoms. Imaging modalities such as CT, MRI, and ultrasonography play a critical role in their diagnosis. The diagnostic sensitivity of ultrasonography for retroperitoneal abscesses ranges from 67% to 87%. CT is considered the most reliable diagnostic tool, with a sensitivity between 90% and 100%. The sensitivity of MRI ranges from 88.5% to 100%. In one case series, CT was found to be less sensitive than MRI for abscesses of spinal origin (67% vs. 100%) [4]. In our case, the definitive diagnosis of the retroperitoneal abscess was made via CT.

According to the literature, retroperitoneal abscesses are most commonly of renal or gastrointestinal origin [5,6]. These infections usually have an insidious course, and morbidity may increase by the time the diagnosis is made. In particular, immunocompromising conditions such as diabetes mellitus predispose patients to bacterial infections [7]. In our patient, who had a 10-year history of type 2 diabetes mellitus, the HbA1c level was 16%, indicating poor long-term glycemic control. Despite no prior history of abdominal pathology, she had recurrent urinary tract infections, most likely representing the initial source of infection. Although urine cultures were negative, this was considered likely due to prior empiric antibiotic use, which may have suppressed bacterial growth.

The causative pathogen in retroperitoneal abscesses varies depending on the source of infection. In a case series, gastrointestinal-source abscesses commonly involved a mix of aerobic and anaerobic bacteria. *Escherichia coli* was the most frequently isolated organism, followed by *Klebsiella* spp. Gram-negative abscesses typically result from ruptured corticomedullary abscesses, whereas *Staphylococcus aureus* infections are more associated with ruptured cortical abscesses [4]. In our case, *Klebsiella pneumoniae* was isolated as the causative organism of the retroperitoneal abscess [8].

Treatment of retroperitoneal abscesses should include both broad-spectrum antibiotics and image-guided drainage. In cases with multiloculated collections, multiple drainage catheters may be required. In our patient, successful management was achieved with percutaneous pigtail catheter drainage and appropriate antibiotic therapy. Although open surgical drainage has traditionally been considered the first-line approach for complex and multiloculated retroperitoneal abscesses, recent evidence supports the use of multistaged percutaneous drainage as a less invasive and effective alternative, especially when combined with culture-directed antibiotic therapy [9]. Adequate glycemic control is essential in patients with diabetes to reduce infection severity and recurrence risk. Furthermore, long-term follow-up is important to monitor for recurrence, which has been reported to be more frequent after open drainage compared to percutaneous approaches. Alfarissi et al. emphasized the high morbidity associated with retroperitoneal abscesses and highlighted the importance of early diagnosis and adequate drainage in their management study [10].

The extraperitoneal location of these abscesses often results in minimal and nonspecific clinical signs. Abdominal pain is typically a late symptom in retroperitoneal abscesses and has been reported in fewer than 50% of cases [11]. These abscesses can extend to adjacent areas such as the thigh, groin, or scrotum. The mean time from symptom onset to diagnosis has been reported to be around 13 days. In our case, the abscess had not extended beyond the abdomen. The patient reported right-sided flank pain three days prior to hospitalization and described intermittent upper back pain during the preceding three months.

## Conclusions

Retroperitoneal abscesses represent challenging infectious conditions, particularly in immunocompromised individuals. The nonspecific nature of clinical symptoms may contribute to delayed diagnosis and increase the risk of complications. For patients with diabetes with recurrent urinary tract infections, radiological evaluation of the retroperitoneal space should be considered an essential component of clinical assessment.

This case illustrates that a multiloculated retroperitoneal abscess caused by *Klebsiella pneumoniae* in a patient with diabetes can be effectively treated with percutaneous drainage under ultrasonographic guidance combined with appropriate antibiotic therapy. It should also be noted that repeat drainage procedures may be required when the initial intervention is insufficient.

In summary, although retroperitoneal abscesses are rare, early diagnosis and a multidisciplinary treatment approach enable successful management. This report emphasizes the importance of considering retroperitoneal abscesses in the differential diagnosis and highlights that timely intervention can significantly improve patient outcomes, especially in those with diabetes. Additionally, while open drainage has traditionally been the first-line treatment for multiloculated retroperitoneal abscesses, our case demonstrates that multistaged or multiple-catheter percutaneous drainage can serve as an effective and less invasive alternative. Given the higher risk of recurrence compared with open drainage, careful long-term follow-up exceeding one year is strongly recommended.
